# Accuracy of Factory-Calibrated Continuous Glucose Monitors in Critically Ill Patients Receiving Intravenous Insulin: A Prospective Clinical Trial of Two Leading Systems

**DOI:** 10.1177/19322968251338865

**Published:** 2025-05-08

**Authors:** Gautam Ramesh, Emily Kobayashi, Navyaa Sharma, Amit R. Majithia, Kristen Kulasa, Schafer C. Boeder

**Affiliations:** 1 Department of Pediatrics, Children’s Hospital of Philadelphia, Philadelphia, PA, USA; 2 Bioinformatics and Systems Biology Graduate Program, University of California, San Diego, La Jolla, CA, USA; 3 Department of Computational and Systems Biology, University of California, Los Angeles, Los Angeles, CA, USA; 4 School of Medicine, Division of Endocrinology & Metabolism, Department of Medicine, University of California, San Diego, La Jolla, CA, USA

**Keywords:** Abbott FreeStyle Libre Pro, continuous glucose monitor (CGM), critical care, Dexcom G6 Pro, mean absolute relative difference (MARD)

## Abstract

**Background:**

Continuous glucose monitors (CGMs) are increasingly being used to guide glucose management in the hospital. However, uncertainty regarding their accuracy in this setting remains.

**Methods:**

We conducted a nonrandomized, open-label, clinically blinded prospective trial of the Dexcom G6 Pro (G6P) and FreeStyle Libre Pro (FLP) in the inpatient setting among critically ill hospitalized patients (n = 40) requiring continuous intravenous insulin infusion. In parallel with CGM data, reference serum (Lab) glucose and point-of-care (POC) glucose values were obtained. On completion of the study, CGM and reference values were analyzed to assess CGM accuracy.

**Results:**

A total of 1015 matched G6P-Lab pairs had a mean absolute relative difference (MARD) of 22.7%, 2369 G6P-POC pairs had an MARD of 22.9%, 1006 matched FLP-Lab pairs had an MARD of 25.2%, and 2353 FLP-POC pairs had an MARD of 27.0%. Both CGM systems demonstrated considerable inter-patient variability in sensor accuracy and tended to underestimate glucose in comparison with the reference values. Rarely were low reference values overestimated by either sensor.

**Conclusions:**

Factory-calibrated continuous glucose monitors may require accuracy validation and per-patient calibration for inpatient use in critically ill patients.

## Introduction

Diabetes is the eighth leading cause of death in the United States and a major risk factor for heart disease (#1), stroke (#5), and kidney disease (#9).^1^ Over 38 million Americans live with diabetes, driving over $400B in annual health care spending.^2^ When critically ill patients with diabetes are hospitalized, multiple factors contribute to dysglycemia, including immune dysregulation, persistent inflammation, and endocrine/metabolic dysfunction.^3^ Poor glycemic control worsens their condition; hyperglycemia increases infection risk and can cause volume imbalance and osmotic insults, while hypoglycemia raises the risk of neurological complications and death. Careful glycemic control improves patient outcomes,^4^ and the American Diabetes Association recommends a target of 140 to 180 mg/dL in critically ill patients with hyperglycemia, with stricter goals when hypoglycemia risk is low.^5^

Continuous glucose monitors (CGMs) are discreet, wearable sensors that measure interstitial glucose, a proxy for serum glucose. While CGM is commonly used in outpatient diabetes management, inpatient glucose monitoring still relies primarily on point-of-care (POC, fingerstick) glucose checks.^5^ In patients already using CGM or automated insulin delivery systems paired with CGM, inpatient CGM with confirmatory POC testing is indicated when appropriate support is available. During the COVID-19 pandemic, many hospitals adopted CGM to reduce nursing exposure and preserve personal protective equipment under a temporary Food and Drug Administration (FDA) policy. The current study aimed to better characterize the accuracy of factory-calibrated CGMs in critically ill patients and determine whether CGM is a viable inpatient glucose monitoring method compared with POC and phlebotomy reference standards.

## Methods

**Figure 1.**
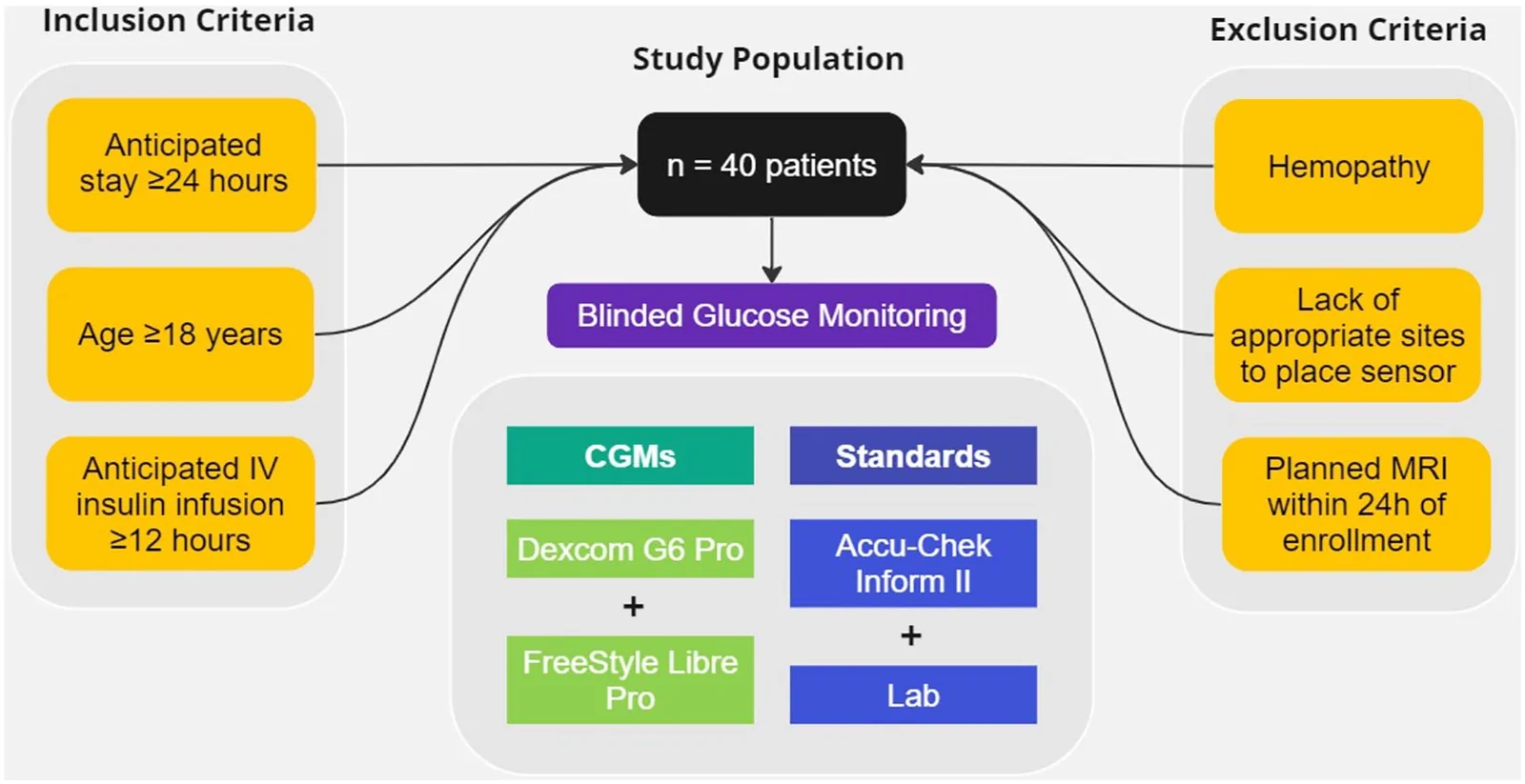
Study population.

We conducted a nonrandomized, open-label, clinically blinded prospective clinical trial (ClinicalTrials.gov ID NCT05081817) of two CGM devices worn simultaneously by medical, surgical, and cardiac intensive care unit (ICU) patients (n = 40) requiring continuous intravenous (IV) insulin infusion.^6^ Eligible participants were ≥18 years old, admitted between November 2019 and December 2021, with an anticipated stay of ≥24 hours and expected need for standard of care IV insulin infusion for at least 12 hours (Figure 1). Exclusion criteria included bleeding disorders, anticoagulant treatment, platelet count <50 000/mL, lack of suitable sensor sites (eg, scars, irritation, wounds, or dressings), scheduled magnetic resonance imaging (MRI) within 24 hours, or if researchers believed participation could jeopardize safety.

The two CGM devices used were the Dexcom G6 Pro (G6P; Dexcom, Inc, San Diego, California) and the FreeStyle Libre Pro (FLP; Abbott Diabetes Care, Alameda, California; Table 1). Both are FDA-approved for outpatient diabetes management. Glucose data were blinded by storing CGM values on sensors for later upload to proprietary servers without local display. Data were not available to clinical staff. The use of CGM posed no added risk beyond standard of care, and all patients gave informed consent, directly or via a surrogate. This study was approved by the University of California, San Diego Institutional Review Board.

**Table 1.** Comparison of Dexcom G6 Pro (G6P) and Freestyle Libre Pro (FLP) CGMs.

Features	G6P^7^	FLP^8,9^
Manufacturer-reported MARD	9.8%	12.3%
Maximum duration of wear	10 days	14 days
Calibration requirement	None	None
Reporting interval	5 minutes	15 minutes
Other notes	Unaffected by acetaminophen up to 1 g every 6 hours. Use of hydroxyurea will lead to blood glucose overestimation.	May be affected by dehydration, and use of ascorbic acid or salicylic acid.
Contraindications	MRI, CT, diathermy, pregnancy, dialysis, critical illness	MRI, CT, diathermy, pregnancy, dialysis, critical illness
Manufacturer recommended anatomic site	Abdomen	Posterior upper arm
Warm-up period	2 hours	1 hour

A research registered nurse (RN) simultaneously placed subcutaneous CGMs on the abdomen or posterior upper arm (n = 9 and n = 31, respectively, G6P), and posterior upper arm (n = 40, FLP). Participants continued standard IV insulin infusion as clinically indicated. Serum glucose measurements (Roche Cobas System hexokinase assay; Roche Diagnostics Indianapolis, Indiana) or POC capillary, venous, or arterial glucose measurement (Roche Accu-Chek Inform II; Roche Diabetes Care GmbH, Basel, Switzerland) were collected as clinically indicated. Additional serum samples were collected every four hours during IV insulin infusion unless a clinical sample was already available. Serum (Lab) reference values were drawn from arterial or venous samples per ICU protocol. Glucose readings from both CGMs were compared with the same reference value. The CGM devices were worn for 10 days or hospital discharge, whichever occurred first. Sensors were removed and reapplied for MRI and shielded for computed tomography (CT) and X-rays unless removal was necessary.

Additional metrics included age, biological sex, diagnoses, comorbidities, complications, and medications (Table 2). Continuous glucose monitor values were time-matched with POC and Lab (serum) values and analyzed for accuracy. Demographic and health data were gathered from the electronic medical record (EMR) and stored on a secure University of California, San Diego server. Continuous glucose monitor data were transmitted to proprietary servers and later downloaded.

**Table 2.** Participant Characteristics.

Characteristic	n	%
Demographics		
Biological sex (female)	11	27.5
Race (white)	12	30.0
Ethnicity (Hispanic, Latino/a, or Spanish origin)	19	47.5
Age (y)		
<45	3	7.5
45-54	8	20.0
55-64	11	27.5
>64	18	45.0
BMI (kg/m^2^)		
>25	34	85.0
>30	23	57.5
Past Medical History		
Diabetes	33	82.5
Hypertension	29	72.5
Hyperlipidemia or dyslipidemia	24	60.0
Coronary artery disease	28	70.0
Heart failure	18	45.0
Chronic obstructive pulmonary disease	4	10.0
Chronic kidney disease	16	40.0
Edema or anasarca	9	22.5
Primary Admission Diagnosis		
Atherosclerotic heart disease, or myocardial infarction	20	50.0
Other cardiac disease	8	20.0
Vascular disease	3	7.5
Sepsis	3	7.5
Kidney disease	1	2.5
Requirements		
Mechanical ventilation	26	65.0
Endotracheal intubation	25	62.5
Hemodialysis	1	2.5
Medications		
Acetaminophen	38	95.0
Aspirin	34	85.0
ACEi or β-blocker	30	75.0
Dopamine (cardiotonic)	25	62.5
Corticosteroids	8	20.0
Vasopressors	34	85.0
Ascorbic acid	1	2.5
Vitals		
SBP <90	14	35.0
DBP <60	28	70.0
SBP <90 and DBP <60	13	32.5
Temperature <97.0°F	19	47.5

Two participants did not declare race. Past Medical History of Diabetes: HbA1c ≥6.5 or prior diagnosis of diabetes. Five participants did not have prehospital HbA1c data, three of whom had diagnoses of type 2 diabetes. Three participants did not have primary admission diagnoses at the time of chart review. Requirements, Medications, and Vitals represent the presence of indicated factors at some point during the admission. Corticosteroids: dexamethasone, fludrocortisone, hydrocortisone, methylprednisolone, or prednisone. Vasopressors: ephedrine, midodrine, norepinephrine, or phenylephrine. ACEi, angiotensin-converting enzyme inhibitor; SBP, systolic blood pressure; DBP, diastolic blood pressure.

Analysis of CGM, POC, and Lab values was conducted using Python 3.8.18.^10^ Preprocessing of CGM included removing missing data and coercion to numeric format. Three CGM profiles with incorrect timestamps were time-shifted. Seven POC values taken within 10 minutes of another POC value were examined; six were excluded as erroneous. A thorough investigation into the POC and Lab “reference values” resulted in an additional seven reference values removed. Each reference value was matched with the nearest CGM value within 15 minutes. Given their native sampling intervals, G6P pairs were always within five minutes, FLP within 15 minutes. For inter-CGM comparison, G6P data were also analyzed using every third reading (to simulate a 15-minute reporting interval). The mean absolute relative difference (MARD) was calculated as previously described.^11^ Time-series plots of each participant’s CGM and reference values were generated using Matplotlib 3.8.0.^12^ Matched pairs were plotted on a Diabetes Technology Society (DTS) Error Grid.^13^ Error Grid zone analysis was performed using the DTS online tool.^14^

## Results

Of the 40 participants enrolled in the study, most were men (Table 2). Over half had a BMI above 30 kg/m^2^ and 33 had diabetes mellitus. Mean CGM wear time was 143 (SD 45) hours for G6P and 140 (SD 44) hours for FLP. Ninety-five percent of participants received acetaminophen, 85% received aspirin, 85% required vasopressors, and only one received ascorbic acid during the study. Fourteen participants had systolic blood pressure below 90 while enrolled in the study, and 19 had temperature below 97°F. Two participants (23 and 26) were admitted with anticipated IV insulin infusion requirement but did not require IV insulin during their hospital stay. Both participants were included in the final analysis per intention-to-treat protocol.

There were 1015 matched G6P-Lab pairs with MARD of 22.7%, 2369 G6P-POC pairs with MARD of 22.9%, 1006 matched FLP-Lab pairs with MARD of 25.2%, and 2353 FLP-POC pairs with MARD of 27.0% (Table 3). The %15/15, %20/20, and %30/30 CGM-reference pair agreement rates are reported in Table 4. The MARDs yielded by matched pairs of G6P values reported every 15 minutes were virtually unchanged from the MARDs of the native five-minute reporting interval (Table 3). The overall median absolute relative difference (ARD), as well as MARD and median ARD within and after the first 24 hours, were also calculated. Marginally lower MARDs were observed for the G6P pairs within the first 24 hours, and higher MARDs were observed for the FLP within the first 24 hours (Table 3). The MARDs were also calculated using the nearest subsequent CGM value to each reference value, as opposed to the absolute nearest (preceding or succeeding), and remained virtually unchanged. In addition, MARDs stratified by anatomic location (abdomen and posterior upper arm) for the G6P were calculated, with mildly higher MARDs for sensors placed on the abdomen than the posterior upper arm. No scatter plot trend was appreciated among participants whose G6P was placed on the abdomen versus posterior upper arm. The MARDs and median ARDs were slightly higher for G6P pairs when off IV insulin infusion compared with when on IV insulin infusion, while the reverse was true for the FLP (Table 3).

**Table 3.** Mean and Median ARD Values.

	Method	n	Mean ARD	Median ARD
G6P^a^	Lab	1015	22.7%	18.1%
	Point-of-Care	2369	22.9%	18.1%
G6P^b^	Lab	1008	22.9%	17.9%
	Point-of-Care	2353	23.0%	18.3%
FLP	Lab	1006	25.2%	24.9%
	Point-of-Care	2353	27.0%	27.0%
G6P, first 24h	Lab	194	21.5%	18.2%
	Point-of-Care	554	22.9%	18.2%
G6P, after 24h	Lab	821	23.0%	18.1%
	Point-of-Care	1815	22.9%	18.0%
FLP, first 24h	Lab	191	32.9%	32.0%
	Point-of-Care	557	30.1%	29.9%
FLP, after 24h	Lab	815	23.4%	23.3%
	Point-of-Care	1796	26.1%	26.3%
G6P, on IV insulin	Lab	565	21.4%	17.2%
	Point-of-Care	1720	22.6%	18.3%
G6P, off IV insulin	Lab	450	24.3%	19.5%
	Point-of-Care	649	23.7%	17.7%
FLP, on IV insulin	Lab	566	28.5%	27.6%
	Point-of-Care	1719	28.2%	28.0%
FLP, off IV insulin	Lab	440	20.9%	20.2%
	Point-of-Care	634	23.8%	24.3%

n: matched pairs; ARD: absolute relative difference; Lab: serum samples; first 24h: data from first 24 hours of CGM wear; after 24h: data after first 24 hours of CGM wear; IV: intravenous.

a Native five-minute sampling interval.

b Subset of Dexcom G6 Pro (G6P) datapoints with virtual 15-minute reporting interval, matched to reference values within 15 minutes, for comparison with FreeStyle Libre Pro (FLP).

**Table 4.** Percent of CGM Pairs Within Specified Range, in mg/dL.

		%15/15	%20/20	%30/30
G6P	Lab	43.3%	55.0%	70.9%
	Point-of-Care	43.0%	54.9%	70.9%
FLP	Lab	20.6%	34.3%	69.2%
	Point-of-Care	18.5%	27.5%	61.7%

Lab: serum samples.

**Figure 2.**
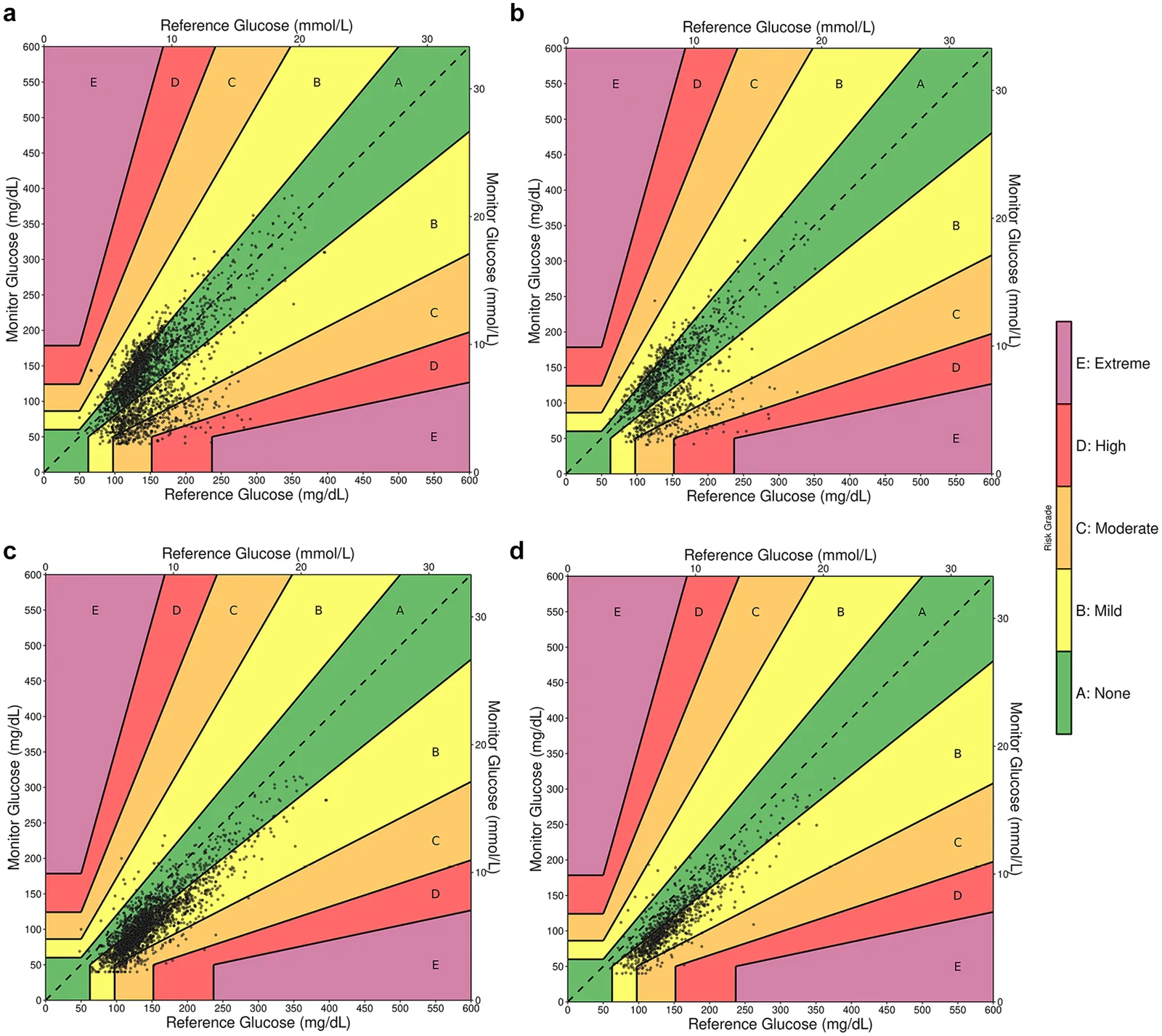
CGM versus reference, diabetes technology society error grids. (a) Dexcom G6 Pro (G6P) versus POC; (b) G6P versus Lab; (c) FreeStyle Libre Pro (FLP) versus POC; (d) FLP versus Lab.

Aggregate data were visualized with Diabetes Technology Society (DTS) Error Grids (Figure 2), as well as Surveillance, Parkes, and Clarke Error Grids (Supplementary Figures 1-3). In analysis across the four common error grid types, 88.6% to 99.8% of values were captured in mild risk and no risk zones (Table 5). Both CGM systems tended to underestimate glucose in comparison with the reference values. In particular, the G6P underestimated reference values when in moderate- to high-risk zones of the Surveillance Error Grid. Also of note, FLP data points consistently clustered below the ideal y = x line but still showed a strong alignment with the reference values. In the DTS, Surveillance, Parkes, and Clarke Error Grid analyses, a greater proportion of FLP values lay in the mild risk zone than the no risk zone. Rarely were low reference values overestimated by either sensor.

**Table 5.** CGM Versus Reference Values by Error Grid Region.

	Risk zone	DTS error grid		Surveillance error grid		Parkes error grid		Clarke error grid		Total
		Count	Frequency	Count	Frequency	Count	Frequency	Count	Frequency	
G6P-POC	A: No Risk	1289	54.4%	1383	58.4%	1359	57.4%	1289	54.4%	2369
	B: Mild Risk	806	34.0%	788	33.3%	934	39.4%	1031	43.5%	
	C: Moderate Risk	251	10.6%	197	8.3%	76	3.2%	10	0.4%	
	D: High Risk	23	1.0%	1	0.0%	0	0.0%	24	1.0%	
	E: Extreme Risk	0	0.0%	0	0.0%	0	0.0%	15	0.6%	
G6P-Lab	A: No Risk	552	54.4%	612	60.3%	581	57.2%	552	54.4%	1015
	B: Mild Risk	355	35.0%	327	32.2%	390	38.4%	435	42.9%	
	C: Moderate Risk	93	9.2%	76	7.5%	44	4.3%	1	0.1%	
	D: High Risk	15	1.5%	0	0.0%	0	0.0%	13	1.3%	
	E: Extreme Risk	0	0.0%	0	0.0%	0	0.0%	14	1.4%	
FLP-POC	A: No Risk	637	27.1%	1150	48.9%	566	24.1%	644	27.4%	2353
	B: Mild Risk	1599	68.0%	1093	46.5%	1778	75.6%	1691	71.9%	
	C: Moderate Risk	113	4.8%	110	4.7%	9	0.4%	2	0.1%	
	D: High Risk	4	0.2%	0	0.0%	0	0.0%	16	0.7%	
	E: Extreme Risk	0	0.0%	0	0.0%	0	0.0%	0	0.0%	
FLP-Lab	A: No Risk	337	33.5%	529	52.6%	280	27.8%	340	33.8%	1006
	B: Mild Risk	640	63.6%	446	44.3%	723	71.9%	662	65.8%	
	C: Moderate Risk	28	2.8%	31	3.1%	3	0.3%	0	0.0%	
	D: High Risk	1	0.1%	0	0.0%	0	0.0%	4	0.4%	
	E: Extreme Risk	0	0.0%	0	0.0%	0	0.0%	0	0.0%	

Count: number of matched-pairs within specified region. Total: total number of matched-pairs. POC: point-of-care blood glucose. Lab: serum blood glucose. DTS: Diabetes Technology Society.

**Figure 3.**
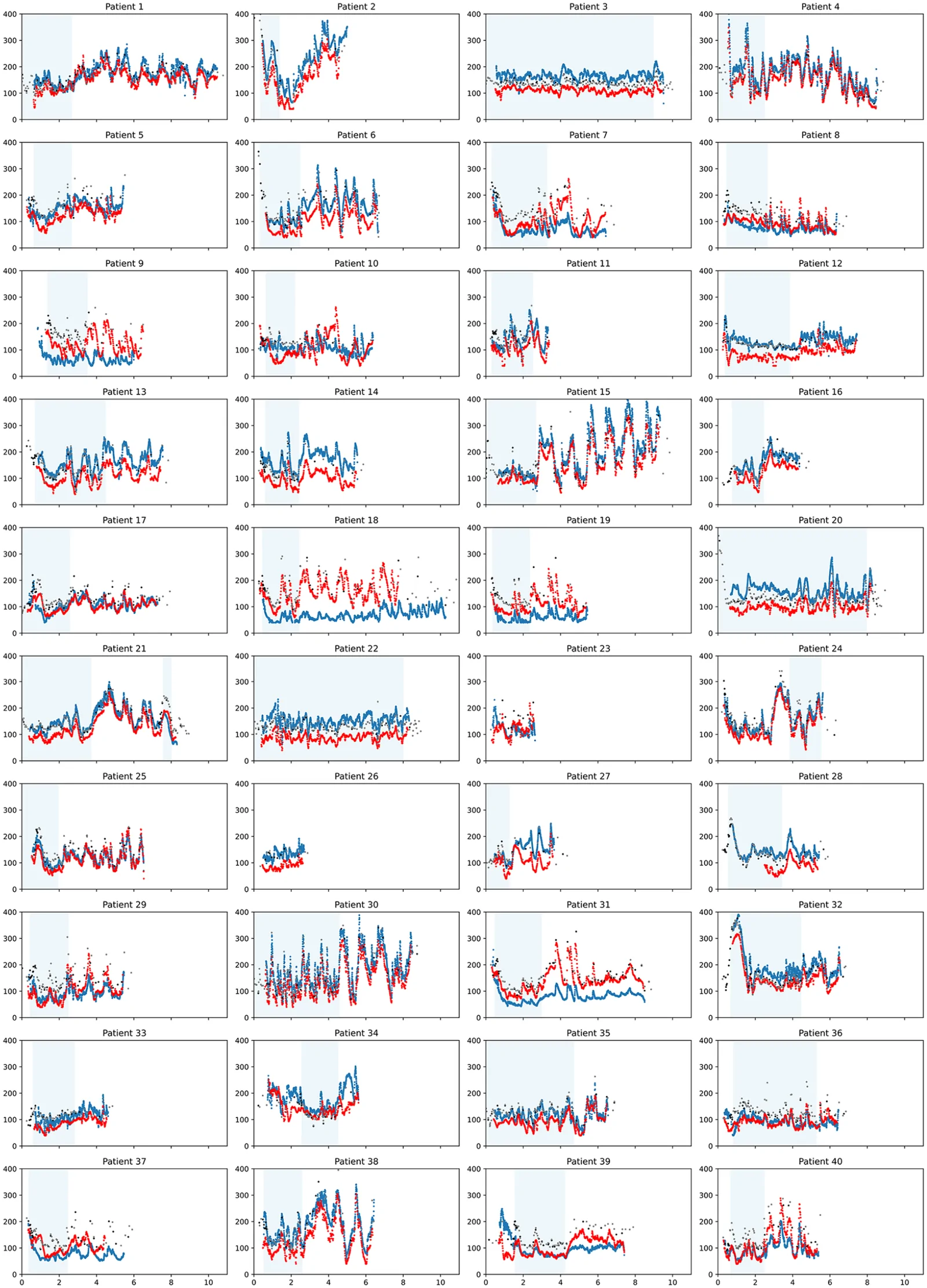
Per-patient time-series scatter plots. Y-axis: glucose (mg/dL), X-axis: time (each tick represents two days). Blue: G6 Pro; Red: FreeStyle Libre Pro (FLP); Gray: Point-of-care; Black: Lab (serum). Light blue shading indicates the time during which insulin was infused.

Per-participant CGM accuracy was visualized using time-series scatter plots (Figure 3), which demonstrate high inter-participant variability. Participant 1 had good concordance between the G6P (blue), FLP (red), and reference (gray and black) values. Twenty-three participants had G6P data that consistently reported higher values than the FLP; in several instances, both CGMs had high visual precision but were consistently either above or below reference values. For example, the G6P worn by participant 3 consistently overestimated glucose values, while the FLP consistently underestimated the glucose. Similar trends were observed for participants 20, 28, 32, and 34. Ten participants had FLP values that consistently reported higher values than the G6P. In nearly all these instances, both the FLP and G6P underestimated glucose when compared with the reference values. In no participants did both devices overestimate glucose values. In a few instances, one or both CGMs did not follow the pattern of reference values, such as the G6P for participant 18, or both devices for participant 36. To better understand the contribution of participant-specific or sensor-specific factors to the MARDs and median ARDs, we calculated the aggregate mean of the MARDs per participant, and likewise for median ARDs. We found lower MARDs and median ARDs, but higher standard deviations, for the G6P pairs when compared with FLP pairs (Table 6). Of participants with individual MARDs higher than the 75th percentile for either device, only two participants (29 and 40) had MARDs above the 75th percentile for both devices.

**Table 6.** Mean and Median ARD Values, and Standard Deviations, Calculated Per-Participant Prior to Aggregation.

	Method	n	Mean ARD^a^	Median ARD^a^	SD^a^
G6P	Lab	1015	23.6%	17.0%	15.8%
	Point-of-Care	2369	24.3%	16.8%	16.3%
FLP	Lab	1006	26.8%	28.0%	7.2%
	Point-of-Care	2353	28.0%	28.0%	8.5%

n: matched pairs; ARD: absolute relative difference; Lab: serum samples.

a Calculated per participant and then aggregated for all participants.

## Discussion

Glycemic control in hospitalized patients with diabetes is crucial. In this prospective trial, we show that factory-calibrated CGMs may not be accurate without additional calibration in critically ill patients requiring insulin infusion. However, our results suggest these systems retain high precision and serve as a promising means of glucose monitoring in patients requiring frequent glucose checks or reduced provider contact (eg, with contagious infections or severe immunodeficiency).

Several trends emerged when analyzing CGM and reference values. Both CGMs frequently underestimated glucose, with the FLP often reading lower than the G6P. The FLP data consistently clustered in the mild-risk region, suggesting high precision but a proportional underestimation of glucose, resulting in elevated MARD and modest purported accuracy. This implies that CGMs can precisely track glucose trends but may require patient-specific calibration for improved accuracy. On the DTS Error Grids, the G6P showed two clusters—one in the no-risk zone, and one in the underestimated moderate-to-high risk zone, possibly representing a contingent of faulty or poorly calibrated sensors. In no participants did both devices overestimate glucose values. This is of clinical benefit given the potentially severe consequences of unrecognized hypoglycemia during insulin therapy. A few participants (eg, participant 36) had CGM values that poorly tracked reference trends. Given the study blinding, this could stem from isolated factory calibration, a faulty unit (typically replaced in clinical practice), or patient-specific factors (eg, interfering medications or conditions). Most participants with individually high (>75th percentile) ARDs in one device did not show high ARDs in the other, suggesting sensor-driven variability plays a greater role than patient-specific factors.

In this critical-care study, most participants required vasopressors and analgesics (eg, acetaminophen, aspirin), and had BMI >30 kg/m^2^—all of which could affect sensor accuracy and contribute to the observed MARDs. This presents an opportunity to innovate next-generation sensors for critically ill patients, particularly those with conditions like edema or hypotension. For the G6P, MARD remained consistent within the first 24 hours of wear compared with after 24 hours. In contrast, the FLP showed higher MARDs during the first 24 hours, improving significantly thereafter. The G6P was more accurate in patients on IV insulin, whereas the FLP performed better in those off IV insulin. While these findings may be incidental, they could also suggest differences in sensor performance across clinical settings.

An increasing number of studies have explored inpatient CGM. One randomized trial found that real-time CGM with Dexcom G6 (DG6) reduced hypoglycemia in insulin-treated patients on general medicine wards.^15^ Another trial evaluating CGM effectiveness in guiding insulin treatment found comparable glycemic control between CGM-guided and POC-guided insulin therapy in general medicine and surgical patients.^16^ One observational study in COVID-19-positive patients in critical care and general medicine floors found DG6 MARDs of 10.9% (vs Lab) and 13.9% (vs POC).^17^ However, the researchers only used CGM data from sensors yielding values within 35 mg/dL of POC in the first 24 hours. Furthermore, Lab glucose was measured only in the morning, and POC glucose checks were only performed in the evening or to confirm CGM values <80 or >400 mg/dL. Our previous unblinded retrospective study of the DG6 in critically ill COVID-19 patients showed improved glycemic control with CGM-directed insulin therapy and MARD of 14.8%.^18^ Sensors were placed on the posterior upper arm, and CGM values were time-matched to reference values within five minutes. The sensors were not calibrated at the bedside but were removed by the care team if inaccurate.

A retrospective analysis of 218 general medicine and surgery patients with diabetes, treated with insulin, found a DG6 MARD of 12.8%.^19^ The MARD was calculated using the CGM value subsequent to each POC reference value—an approach that partially accounts for the inherent lag in interstitial glucose measurements but is less conservative than using the nearest CGM value. This may reduce the clinical relevance of the MARD. Thus, we chose to use the nearest CGM value to each reference without applying any lag time correction.

In one observational study, ICU patients with factory-calibrated DG6 sensors had an MARD of 13.19%, while those with additional calibration at two, 12, and 24 hours had an MARD of 9.42%.^20^ Although accuracy was good with both factory-calibrated and additionally calibrated sensors, data from patients with MARD >25% were excluded. Other studies have evaluated blinded CGMs without validation or calibration. One reported a G6P MARD of 19.2% in noncritical patients^21^; another found an FLP MARD of 14.8%.^22^ Our G6P MARDs (22.9% vs POC, 22.7% vs Lab) align with the former. The higher FLP MARD in our study may reflect the inclusion of critically ill patients. This is the first prospective clinical trial to compare two factory-calibrated CGMs, side-by-side, in critically ill patients. Using professional-use CGMs enabled blinded monitoring and robust analysis. Notably, both Dexcom and Abbott have released newer CGMs since the inception of this study—Dexcom G7 and the FreeStyle Libre 3, respectively—which are being studied in hospitals and may address some of the issues highlighted here.

This study has several limitations. Blinding CGM data to both clinical and research teams prevented bedside calibration and replacement of inaccurate or defective sensors. This likely reduced the accuracy, but not the precision, of the CGMs. Nevertheless, the high MARDs underscore the need for further evaluation in critically ill patients. Thirty-one participants wore the G6P on the upper arm rather than the manufacturer-recommended site (abdomen). This did not appreciably affect MARDs, but further study of alternate sites is warranted. Our modest sample size (n = 40) from a single site may limit generalizability. Larger studies stratifying by confounders (eg, patient metrics, medications)^23^ are needed to clarify their impact on CGM performance in critically ill patients.

We believe CGM use in the hospital offers many potential benefits: reduced workload for bedside staff, improved isolation compliance, decreased personal protective equipment usage, reduced hypoglycemia, and possibly improved glycemia. Furthermore, CGM could enable more precise titration of insulin or glucose infusion in conditions like diabetic ketoacidosis, hyperosmolar hyperglycemic state, stress- or steroid-induced hyperglycemia, or hyperinsulinism. For example, one randomized trial found increased time in euglycemia and reduced hypoglycemia in very preterm infants with CGM-guided glucose titration.^24^ With further study and optimization, CGM may become standard tools in inpatient care, enhancing resource use and patient outcomes.

## Conclusion

Continuous glucose monitors show promise for inpatient use. However, factory-calibrated CGMs may require accuracy validation and additional bedside calibration to ensure optimal performance, particularly in critically ill patients. Further trials are needed to refine inpatient CGM protocols and better understand their accuracy in this population.

## Supplemental Material

sj-docx-1-dst-10.1177_19322968251338865 – Supplemental material for Accuracy of Factory-Calibrated Continuous Glucose Monitors in Critically Ill Patients Receiving Intravenous Insulin: A Prospective Clinical Trial of Two Leading Systems Supplemental material, sj-docx-1-dst-10.1177_19322968251338865 for Accuracy of Factory-Calibrated Continuous Glucose Monitors in Critically Ill Patients Receiving Intravenous Insulin: A Prospective Clinical Trial of Two Leading Systems by Gautam Ramesh, Emily Kobayashi, Navyaa Sharma, Amit R. Majithia, Kristen Kulasa and Schafer C. Boeder in Journal of Diabetes Science and Technology
