# Supplementary material for: Accuracy of Factory-Calibrated Continuous Glucose Monitors in Critically Ill Patients Receiving Intravenous Insulin: A Prospective Clinical Trial of Two Leading Systems
Source: J Diabetes Sci Technol. 2025 May 8;20(5):1585–94. doi: 10.1177/19322968251338865 (PMC12061911; doi:10.1177/19322968251338865)
Supplement: Supplementary file 1 [file sj-docx-1-dst-10.1177_19322968251338865.docx]

**Supplementary Material**

**Accuracy of Factory-Calibrated Continuous Glucose Monitors in Critically Ill Patients Receiving Intravenous Insulin: A Prospective Clinical Trial of Two Leading Systems**

Gautam Ramesh, MD, MPH, Emily Kobayashi, BS, Navyaa Sharma, Amit R. Majithia, MD, Kristen Kulasa, MD, Schafer C. Boeder, MD

**Supplementary Figure 1**. **CGM vs. Reference, Surveillance Error Grids**. (A) Dexcom G6 Pro vs. POC; (B) Dexcom G6 Pro vs. Lab; (C) Abbott FreeStyle Libre Pro vs. POC; (D) Abbott FreeStyle Libre Pro vs. Lab.

**Supplementary Figure 2**. **CGM vs. Reference, Parkes Error Grids**. (A) Dexcom G6 Pro vs. POC; (B) Dexcom G6 Pro vs. Lab; (C) Abbott FreeStyle Libre Pro vs. POC; (D) Abbott FreeStyle Libre Pro vs. Lab.

**Supplementary Figure 3**. **CGM vs. Reference, Clarke Error Grids**. (A) Dexcom G6 Pro vs. POC; (B) Dexcom G6 Pro vs. Lab; (C) Abbott FreeStyle Libre Pro vs. POC; (D) Abbott FreeStyle Libre Pro vs. Lab.
